# Increasing Specificity of Targeted DNA Methylation Editing by Non-Enzymatic CRISPR/dCas9-Based Steric Hindrance

**DOI:** 10.3390/biomedicines11051238

**Published:** 2023-04-22

**Authors:** Daniel M. Sapozhnikov, Moshe Szyf

**Affiliations:** Department of Pharmacology and Therapeutics, Faculty of Medicine and Health Sciences, McGill University, Montreal, QC H3G 1Y6, Canada; daniel.sapozhnikov@mail.mcgill.ca

**Keywords:** epigenetic editing, DNA methylation, CRISPR/Cas9, dCas9, DNMT1, DNMT3A, TET1, steric hindrance, demethylation

## Abstract

As advances in genome engineering inch the technology towards wider clinical use—slowed by technical and ethical hurdles—a newer offshoot, termed “epigenome engineering”, offers the ability to correct disease-causing changes in the DNA without changing its sequence and, thus, without some of the unfavorable correlates of doing so. In this review, we note some of the shortcomings of epigenetic editing technology—specifically the risks involved in the introduction of epigenetic enzymes—and highlight an alternative epigenetic editing strategy using physical occlusion to modify epigenetic marks at target sites without a requirement for any epigenetic enzyme. This may prove to be a safer alternative for more specific epigenetic editing.

## 1. Introduction

Gene function is determined by an interaction between DNA sequences and their programming by epigenetic processes. The same gene is programmed to be expressed differently in different cellular, spatial, and temporal contexts by a combination of biological processes that alter the histone proteins in chromatin by a gamut of modifications [1,2,3,4], including acetylation, methylation, phosphorylation [5], ubiquitination [6], as well as the DNA molecule itself by enzymatic methylation of the cytosine base [7] by DNA methyltransferases (DNMTs) (predominantly at the dinucleotide sequence CG), which could be followed by a sequence of oxidations of the methyl moiety by TET enzymes [8].

Disruptions to gene function could occur due to changes to the primary nucleotide sequence, which could be corrected through “genetic editing”, as well as by epigenetic dysregulation, which could potentially be corrected through “epigenetic editing”. The fundamental difference between epigenetic programming and genetic alterations is that epigenetic programming is reversible, and “correction” of an epigenetic program does not require alterations of the integrity of the DNA sequence and is, thus, more appealing as a therapeutic strategy than gene editing.

Most studies examining the relationship between epigenetic modifications and gene function are based on associations between various epigenetic modifications and cellular, physiological, and pathological states. Initially, studies focused on the associations between the epigenetic states of candidate genes and gene expression [9,10]. With the advent of genome-wide methodologies, it became possible to overlay full transcriptomes with genome-wide landscapes of multiple epigenetic modifications [11,12].

Pharmacological inhibitors of epigenetic enzymes [13,14] and knockdown/knockout of genes encoding epigenetic enzymes [15,16,17,18] have established a general causal role for epigenetic modifications in cellular differentiation, physiology, and pathology. However, establishing causation between a specific epigenetic modification, gene expression, and the phenotype has been challenging without methods that could alter an epigenetic modification at a unique genomic position. When general inhibition or depletion of an epigenetic enzyme is used to establish causation, other confounders may also be involved. Epigenetic proteins could have multiple roles in gene regulation as well as additional indirect actions through disrupting cellular homeostasis. Since alterations in epigenetic modifications are implicated in both normal physiology and disease, it is crucial to understand their causal role in cellular processes. From a practical standpoint, the identification of critical modifications is a prerequisite for the rational design of therapeutics aimed at reversing these modifications to cure, prevent disease, or for the improvement of agricultural and biotechnological processes. Epigenetic editing could potentially address this challenge.

Epigenetic editing can target either histone or DNA modifications. DNA modifications are base specific and can provide higher sequence-specific resolution than histone modifications, which can cover a wider region. There is a significant body of evidence that demonstrates DNA methylation alterations in various diseases, such as cancer [19,20,21,22], autoimmune disease [23,24], cardiovascular disease [25,26,27,28], and metabolic disease [24,29]. If it is indeed demonstrated that specific alterations are causal, targeted epigenetic editing could potentially reverse the disease-associated DNA methylation profiles in vivo; in other words, targeted epigenetic editing is well suited to address both causality and correction. Targeted epigenetic programming may also have applications in cell therapy and immune therapy, as in, for instance, trans-differentiation of liver cells to produce insulin through demethylation of the insulin gene and pancreatic-specific transcription factors [28,30] or silencing of checkpoint inhibitors in patient T cells [31,32] to enhance immunotherapy. However, there are still numerous barriers to overcome.

While epigenetic editing for testing causality is already widely practiced [33], there are two major obstacles currently delaying the clinical utility of targeted epigenetic engineering technologies: (1) difficulties in the delivery of CRISPR/Cas9 components to target cells and (2) molecular technologies that fail to achieve specific and efficient epigenetic editing. In this review, we focus on the latter issue by highlighting the disadvantages of using CRISPR/dCas9 systems that involve epigenetic enzymes and present, instead, an argument for the increased clinical applicability of a recently developed epigenetic engineering method based on the steric hindrance of a nuclease-dead Cas9 (dCas9) which simply interferes with endogenous DNA methylation machinery and reduces unwanted epigenetic side effects by foregoing the need for any enzyme as a component of the technology.

## 2. Targeting Architecture of Epigenetic Editors

A tool for site-specific epigenetic editing typically must consist of two components: an enzymatic component with epigenetic-modifying activity (with the exception of targeted DNA methylation editing [34,35], discussed below in the section titled “Steric inhibition for DNA demethylation”) and a targeting component—a domain that can bind a specific DNA sequence so that the epigenetic activity can be targeted to specific genes or genomic locations. At present, targeting is achieved by one of three categories of targeting domains: zinc-fingers (ZFs), transcription activator-like effectors (TALEs), or CRISPR/dCas9. The benefits and drawbacks of each targeting system for epigenetic editing have been extensively reviewed [36], but for research purposes, CRISPR/dCas9 remains by far the simplest to re-target—requiring only a change of the ~20-bp targeting (gRNA) sequence rather than more complicated de novo protein design required for TALEs or ZFs—and, thus, the epigenetic editors discussed in this review will be limited to those that utilize CRISPR/dCas9 as the targeting component. It is important to add that while this ease of use has resulted in the widespread adoption and preference for CRISPR/dCas9-based architecture in the research laboratory setting, TALEs and zinc-fingers remain highly useful in the clinic, where the time-consuming development of a custom protein that targets a single locus is acceptable when the therapeutic goal is the manipulation of a single known disease-causing gene or locus [37,38].

## 3. Enzymatic Epigenetic Engineering for Targeted Methylation and Its Shortcomings

Currently, the enzymatic component of any epigenetic editor for targeted DNA methylation must be a DNA methyltransferase—an enzyme capable of catalytic addition of methyl groups to DNA. Targeted DNA methylation in the mammalian genome using CRISPR/dCas9 was first demonstrated in 2016 by four independent groups: all four approaches were identical in their reliance on the fusion of dCas9 to the DNA methyltransferase DNMT3A (or its catalytic domain) [39,40,41,42]. Though these studies provided an excellent proof-of-principle, the major downside was that efficient methylation required prolonged high expression of dCas9-DNMT3A and declined rapidly after the termination of dCas9-DNMT3A expression. In 2017, two studies reported increased efficiencies of targeted methylation either by the addition of the DNMT3A-stimulating protein DNMT3L as part of the methylation effector domain [43] or by replacing DNMT3A entirely with the potent bacterial methyltransferase M.SssI [44].

While complete and persistent targeted methylation continues to be elusive, the considerably more serious drawback underlying all targeted methylation strategies is the pervasive off-target activity of the methyltransferase effector domains which are overexpressed in all such strategies and are capable of nonspecific methylation independent of the targeted dCas9 that they are fused to. There is no strategy to date that could limit a CRISPR/dCas9 targeted enzyme only to its specific target. The DNMT3A active site must recognize the CG dinucleotide sequence and it is, therefore, impossible to disentangle methyl transfer from CG binding; DNMT3A could, therefore, potentially bind CGs across the genome. The observation that methyltransferases possess a nonspecific activity independent of targeting that renders them inadequate for precise epigenetic editing has been widely accepted since before the development of CRISPR/dCas9 technology [45] and has continued to be consistently demonstrated to be the case. Even in the least efficient targeted methylation strategies—fusions of the catalytic domain of DNMT3A to dCas9 (dCas9-DNMT3A-CD)—the expression of dCas9-DNMT3A-CD causes global methylation changes in cells that are highly similar to those observed upon the expression of DNMT3A alone [46]. dCas9-M.SssI exhibits a similar and potent nonspecific activity [44].

There have been numerous efforts to improve specificity. Two independent groups reported similar strategies which involved activity-reducing point mutations in the DNMT3A (R887E) [47] or M.SssI (Q147L) [44] components of dCas9-based epigenetic editors, both claiming increased methylation specificity. For DNMT3A (R887E), off-target methylation, though reduced, was still detectable; targeted analysis of an arbitrary off-target region in the VEGFA promoter consistently revealed gRNA-independent off-targeted methylation of the R887E mutant, suggesting that the accompanying genome-wide methylation (MBD-seq) analyses (which also reported mild off-target methylation) might be underpowered and underestimate the off-target methylation events that might be detected with a more powerful genome-wide DNA methylation analysis method. For M.SssI (Q147L), the authors analyzed candidate gRNA off-target sites of the gRNA and reported no off-target methylation—yet, this off-target analysis strategy is insufficient in that it does not assess the aforementioned off-target methylation independent of dCas9 by the methyltransferase domain as has been extensively demonstrated. Though the authors also performed reduced-representation bisulfite sequencing, they correctly conclude that a failure to detect off-target effects with these approaches does not equate to an absence of off-target effects and, indeed, a more recent study presented evidence that the Q147L mutation in M.SssI does not reduce its nonspecific activity [48], instead only reducing its catalytic activity which suggests that in the original study, the Q147L mutation leads to off-target methylation that is below the threshold of detection. Off-target detection is highly dependent on the power and comprehensiveness of the detection method; it is likely that deep whole-genome methylation sequencing—which was not used in either study—would reveal the true extent of off-target methylation of these engineered epigenetic editor variants.

Another approach reported a reduction of the nonspecific methylation by recruitment of multiple DNMT3A domains by the SunTag system rather than direct fusion to dCas9 [49,50]. Still, this strategy continues to require overexpression of the methyltransferase domain and nonspecific methylation was still reported. Furthermore, contradictory results from an independent group reported no observed increase in the specificity of the SunTag approach [47].

A final strategy to reduce off-target methylation of epigenetic editors is to split the methyltransferase domain into two components such that the complete methyltransferase domain is reconstituted only at the targeted site (either by targeting the two split domains separately or by targeting one of the split domains to the target site and constitutively expressing, without any targeting, the other split domain). Though there is some evidence that this split methyltransferase approach improves non-specificity compared to dCas9-DNMT3A fusion proteins, the data are limited to only a few targeted sites and lacks whole-genome methylation analysis [51]. Moreover, there are contradictory reports which suggest that split methyltransferase strategies are incapable of targeted methylation as they exhibit similar efficiencies in the methylation of target and non-target sites [45]. Off-target methylation activity, thus, appears to be an inevitable correlate of targeted methylation as long as epigenetic editors remain invariably dependent on the overexpression of methyltransferase domains.

An additional point to keep in mind regarding dCas9-methyltransferase fusions is that human methyltransferases have evolved to interact with dozens of nuclear proteins to regulate transcription which could lead to unintended consequences of the epigenetic editor both at the target site and throughout the genome. DNMT3A, for example, interacts with EZH2 [52], p53 [53], and many other proteins. These interactions are likely to persist even when only the catalytic domain of DNMT3A is used but are likely less relevant for epigenetic editors relying on the non-human methyltransferase M.SssI.

Exceptionally, a new approach to induce methylation in an unmethylated promoter without using enzymes involves the disruption of an unmethylated CG island by the integration of a fragment of CG-less DNA into embryonic stem cells using CRISPR-mediated targeting and recombination [54]. This method triggers methylation of the disrupted CG island and, after inducing methylation, the CG-less fragment is removed by Cre-Lox or Piggybac transposase-mediated recombination. The introduced methylation can be stable and trans-generationally heritable in mice with resulting changes in gene expression and phenotype [55]. While this method is valuable for research, its clinical utility may be limited to modulating cellular therapy due to the high de novo methylation activity required, which is mainly found in embryonic stem cells. Furthermore, this approach can only induce regional methylation, not site-specific methylation. Finally, in its current form, the technique introduces genetic changes that arise from Piggybac transposase-mediated recombination and also risks additional mutations caused by the use of catalytically active CRISPR/Cas9 which may introduce off-target edits [56] and genomic alterations [57] that could confound interpretation of results and limit its clinical utility.

## 4. Enzymatic Epigenetic Engineering for Targeted Demethylation and Its Shortcomings

**Figure 1:**
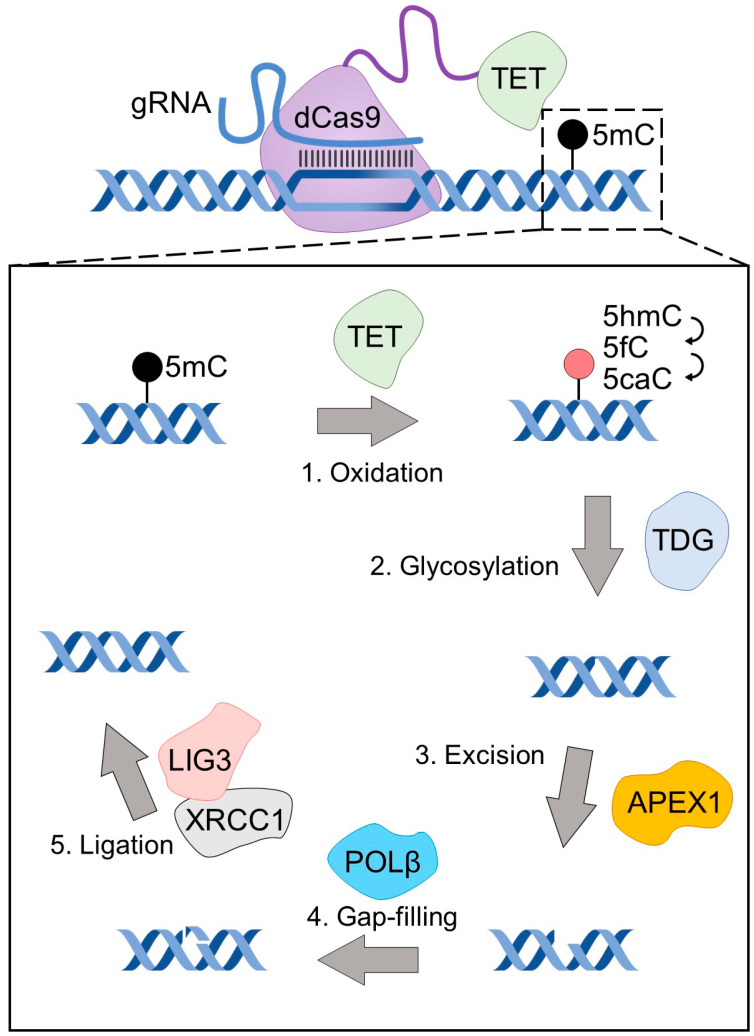
DNA demethylation by dCas9-TET. A schematic diagram of dCas9-TET-based targeted DNA demethylation, highlighting the numerous steps and enzymes required for a methylated CG to be converted to an unmethylated CG by this epigenetic editing tool.

The characteristics of targeted enzymatic DNA demethylation techniques parallel those of techniques for targeted methylation. In humans, active DNA demethylation is initiated by the TET family of enzymes which catalyze the conversion of methylated cytosines to a series of more oxidized forms that are eventually excised from the genome and replaced by unmodified/unmethylated cytosines by DNA repair machinery [58]. Therefore, though TET proteins are not biochemically demethylases, they are classically used as the enzymatic component of CRISPR/dCas9-based targeted demethylation techniques (Figure 1).

In 2016, four independent groups developed CRISPR/dCas9-based systems for targeted DNA demethylation; those that fuse [41,59] or recruit [60,61] the catalytic domain of TET1 with CRISPR/dCas9. Interestingly, despite the ability of all three TET family proteins (TET1, TET2, and TET3) to oxidize methylated CGs [62], fusions of dCas9 to the catalytic domain of TET2 and TET3 have only been presented in one thesis [63] and one article [64], respectively. The general absence of other known efficient demethylases from humans or other organisms has resulted in comparatively reduced innovation in the demethylation editing field (compared to methylation editing which is described above) and has yielded a much simpler landscape of epigenetic editing tools for targeted demethylation than those for methylation. However, there is one strategy for targeted demethylation that does not invoke TET enzymes: ROS1, a glycosylase from *Arabidopsis*, is able to directly initiate the replacement of methylated CGs without the need for an initial oxidation step. To this end, dCas9-ROS1 [65] has been successfully used to demethylate CGs by way of direct glycosylation.

Unlike the tools for targeted DNA methylation, there have been no systematic comparisons of CRISPR/dCas9-based demethylation tools. In a recent study, we tested the demethylation, gene activation, and off-target effects of dCas9-TET targeted demethylation [35]. dCas9-TET effectively demethylated the target (a methylated mouse *Il33* promoter in NIH-3T3 cells) with the highest demethylation directly at the gRNA binding site and activated transcription severalfold over the control. However, similar activation was achieved by a catalytic mutant of TET which, as expected, did not trigger demethylation [35]; gene activation by a catalytic mutant of TET has been previously reported [66]. We further found that TET proteins could increase transcription from a completely unmethylated transfected promotor-reporter. It was also previously reported that the transcriptional effects of TET depletion in cells without all three DNMTs are similar to those in wild-type cells, suggesting significant methylation independent activities of TET [8]. Similarly, TET1 was shown to regulate H3K27 modification independent of its catalytic activity as a catalytic TET1 mutant expressed in embryonic stem cells or mice restored the normal pattern of H3K27me3 and normal differentiation which were deregulated in a TET-/- mutant; TET deficiency causes aberrant upregulation of (H3K4me3+; H3K27me3+) bivalent marks on developmental genes which results in developmental delays [67].

Furthermore, effective activation of a distal promoter of *Il33* was achieved when dCas9-TET was targeted to the proximal promoter as well as in cells expressing a non-targeted (scrambled gRNA) alongside dCas9-TET, suggesting efficient off-target effects by dCas9-TET. This was confirmed with genome-wide bisulfite sequencing which revealed multiple demethylation events across the genome by a dCas9-TET vector targeted to the proximal promoter of the Il33 gene [35]. Off-target effects of dCas9-TET were reported independently [68]. Epigenetic editing with a tethered TET enzyme is, therefore, confounded by (1) the methylation-independent activities of TET as seen by the efficient gene activation with a catalytic TET mutant and its established DNA methylation-independent transcriptional and histone modification effects, (2) the genome-wide untargeted effects of the tethered TET enzyme, and (3) the fact that TET proteins are dioxygenases and not demethylases and, therefore, it is difficult to discriminate whether epigenetic effects are caused by newly demethylated or newly oxidized CGs which impact transcription through numerous gains or losses of interactions [69]. Moreover, TET-mediated demethylation requires base excision repair by TDG and other proteins which might compromise the integrity of the DNA and possibly indirectly affect gene expression. Therefore, TET-based epigenetic editing tools—in contrast to DNMT-based epigenetic tools, which are methylating enzymes—are further confounded by the fact that they bear an enzymatic activity that is not directly demethylating the DNA.

## 5. Steric Inhibition for DNA Demethylation

The specificity of any drug is a fundamental requirement in pharmacology and molecular therapy. Since dCas9-TET as an epigenetic editing tool is confounded at multiple levels, it cannot fully address the mechanistic role of DNA methylation or be used as a specific demethylating agent. In addition to these limitations, any epigenetic editing tool that consists of an enzyme tethered to dCas9 would modify a region of DNA of varying sizes based on processivity, the steric parameters, chromatin structure, and the flexibility of the tethered enzyme and it, therefore, cannot be targeted to a specific CG.

**Figure 2:**
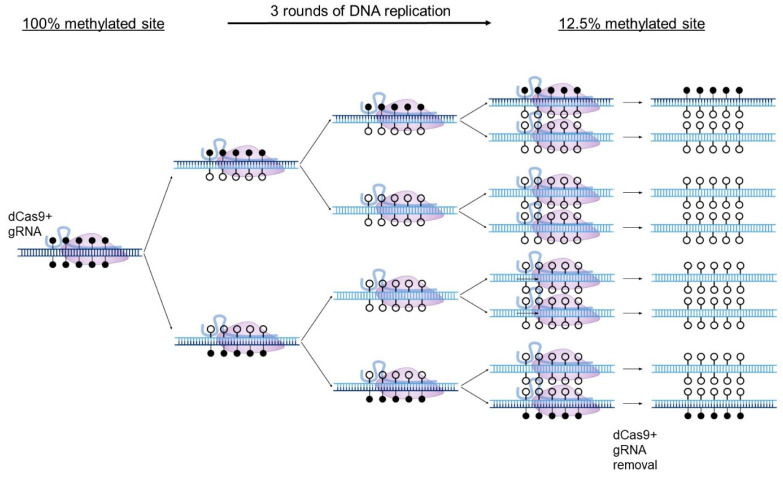
DNA demethylation by dCas9 steric hindrance. A schematic diagram depicting the targeted DNA demethylation induced by the steric hindrance method in dividing cells. Parental DNA strands, shown in dark blue, are diluted as the cells divide. In the presence of a fully effective targeted interference of dCas9 with DNMT1, after 3 rounds of cell division wherein methylation levels halve with every round due to a lack of methylation of nascent DNA strands (light blue), a target site which was originally 100% methylated would be effectively demethylated to 12.5%, though, in practice, more rounds of cell division should be included to further reduce methylation. dCas9 and gRNA expression can then be terminated, leaving the unmethylated target site exposed to potential interacting proteins in the nucleus.

In a recent study [35], we noticed that while the gRNA-guided dCas9-TET vectors caused variable demethylation at CG positions away from their binding sites, the CG positions that were within or proximal to the gRNA binding sites were invariably highly demethylated. This raised the possibility that dCas9 binding alone, irrespective of the tethered enzyme, can cause demethylation by steric hindrance of DNMT: tight binding of dCas9 and its guide RNA to the target CG might be occluding access to DNA methylating machinery and also, possibly, to demethylation machinery. DNA methylation in dividing cells is amenable to steric hindrance-driven demethylation since new unmethylated DNA is generated with each round of DNA replication and the deposition of paternal strand DNA patterns requires faithful methylation by DNMT1. Thus, restricting access of DNMT1 to a small region by a tightly bound dCas9 and gRNA could prevent newly synthesized DNA from acquiring the paternal strand pattern of methylation at the site without affecting the ability of DNMT1 to faithfully replicate methylation elsewhere. After several rounds of replication, the ancestral strands are diluted, and the blocked site becomes demethylated in the population (Figure 2). Steric hindrance by binding of transcription factors had been proposed almost four decades ago as a mechanism for demethylation during development [7].

If dCas9-gRNA binding can indeed block DNMT access to DNA, it could cause demethylation without requiring an additional tethered enzyme, potentially avoiding the confounders discussed above. dCas9 can act as a classic antagonist of DNMT by obstructing the enzyme’s access to the substrate. Compared to a tethered enzyme, steric hindrance is expected to be limited to the CG positions that are targeted and bound to dCas9, resulting in higher specificity. Furthermore, since steric hindrance requires a tight association of dCas9 to its target, weaker binding at off-targets should preclude off-target effects.

To determine whether targeted dCas9 blocks DNA methylation in vitro and to determine the specificity of inhibition, we examined the demethylation of a sequence of DNA that had several CG sites, each targeted by a specific gRNA [35]. The experiment demonstrated potent site-specific inhibition (only the CGs targeted by their specific gRNAs were “demethylated”) of DNA methyltransferase M.SssI when the DNA was preincubated with dCas9 and gRNA. As expected from a steric inhibitor with no demethylase activity, dCas9 had no effect if added to a methylated substrate. In vitro assays of a dense CG island of the p16 gene defined the minimum footprint of dCas9 to be a maximum of 45 base pairs, including the 20-bp gRNA, with a peak inhibition directly within the gRNA site. Moreover, careful positioning of dCas9 in the context of relatively low CG density could be used to protect single CpGs from methylation [35].

The use of in vitro methylated reporter vectors, such as luciferase, to define the functional role of DNA methylation in the silencing of promoters and enhancers has been a standard practice [70]. However, a major challenge of these studies is the difficulty of recapitulating the site-specific methylation profile observed in vivo. While such studies measure the effects of regional methylation on promoter activity, it is unfeasible to use this approach to measure the effects of site-specific methylation events since in vitro methylation has been unable to be performed in a site-specific manner.

However, using this system, it should be possible to selectively inhibit methylation at specific CG positions during in vitro methylation and generate constructs with different demethylated/methylated positions to map the specific methylation events that block transcription. By transiently transfecting these promoter/reporter constructs and measuring their expression activity, it is possible to investigate the effects of site-specific methylation events. Applying this method to the promoter of the mouse *Il33* gene revealed that methylation of three CGs located directly adjacent to the transcription start site (TSS) inhibited the expression of the tested construct, while the methylation of other CGs in the promoter was inconsequential [35]. This experiment illustrates the significance of examining site specific rather than regional methylation.

To test the method in living cells, lentiviral vectors were utilized to express dCas9 and specific gRNAs with all gRNAs directing CG position-specific demethylation. The extent of demethylation was proportional to the gRNA expression level and selecting highly expressed gRNAs resulted in almost completely demethylated cell lines [35]. After demethylation, it is crucial to remove dCas9-gRNA since its binding also blocks interactions between the transcription machinery and the gene. Importantly, gRNA-dCas9 targeting the three CGs located at the transcription start site (TSS) severely impedes transcription, even after these CGs become demethylated [35]. In this study, a Cre-lox strategy was employed by using Floxed dCas9 which was removed after demethylation by expressing Cre recombinase.

After site-specific demethylation and removal of dCas9, we evaluated whether gene activation could be achieved [35]. Our findings indicate that activation of the IL33 gene through site-specific demethylation was comparable to the activation achieved by treating cells with the global DNA methylation inhibitor 5-aza-2′-deoxycytidine, but one order of magnitude smaller than the activation achieved with dCas9-TET epigenetic editing. These results suggest that while steric hindrance-triggered demethylation reveals the specific role of DNA methylation, the effects achieved by dCas9-TET may reflect both DNA methylation-dependent and independent activities, making it difficult to discern the specific contribution of DNA methylation to silencing or the role of specific sites.

Site-specific demethylation with the steric hindrance of other promoters yielded similar results, indicating a small contribution of DNA demethylation itself to gene activation, with one exception being the methylated repeat of the FMR1 gene, where demethylation had a significant impact on gene expression [35]. The findings of these studies highlight the importance of site-specific demethylation in understanding the functional role of DNA methylation at specific sites, as opposed to relying solely on methods of epigenetic reprogramming by dCas9-TET which induces broader epigenetic changes across larger regions. A genome-wide analysis of the potential off-target methylation effects of the gRNAs used in this study showed a significantly higher incidence of off-target demethylation with the use of dCas9-TET compared to dCas9 steric hindrance [35]. These findings support the fundamental premise of the approach which is that excluding a tethered epigenetic enzyme can prevent widespread off-target epigenetic reprogramming.

Initially, the steric hindrance method provides a feasible approach for demethylation in dividing cells and is adaptable to a research setting. However, for the method to be used in an in vivo setting or for cell and in vivo therapy, advances in methods for delivering dCas9-gRNA particles—as protein-RNA or RNA particles—will be necessary. Progress in in vivo delivery of other CRISPR/Cas9-based methods—which is a burgeoning field with a variety of promising new advances [71,72,73,74]—would also benefit the clinical development of the steric hindrance method. Since dCas9 must be removed to enable transcription, methods that do not require stable DNA transfer and deliver proteins or RNA with a shorter half life are preferred. The kinetics of delivery of dCas9 protein-gRNA complexes or dCas9-gRNA RNA could be optimized to achieve the highest blockage of DNA methylation before the concentration of the complexes is reduced and interaction with the transcription machinery is enabled.

The steric hindrance method as presented thus far has a limitation in that it requires DNA replication to induce the loss of DNA methylation which makes it ineffective in non-dividing cells. As a result, the applicability of this method is largely restricted to dividing tissues, ex vivo cellular therapy, and differentiation of progenitor cells derived from induced pluripotent stem cells. Additionally, it is unclear how stable the loss of methylation is and whether it can be protected from de novo methylation after dCas9 removal. While our initial study found stable demethylation, future research is needed to assess the long-term stability of the demethylated state in different cell types and explore potential strategies to maintain the demethylated state [35]. It is worth noting that the effectiveness of steric hindrance in inhibiting methylation may vary depending on the gene and cellular context. Nonetheless, steric hindrance may have the potential for investigating dynamic de novo methylation and demethylation in non-dividing cells by inhibiting, for example, methylation in response to neuronal activation [75,76,77] or behavioral exposure as we will discuss below.

## 6. Steric Inhibition to Either Prevent or Preserve DNA Methylation

Dynamic DNA methylation systems in non-dividing cells, such as neurons, involve both de novo methylation and demethylation events in the absence of DNA replication. De novo methylation is catalyzed by de novo methyltransferases, such as DNMT3A [66,78,79]. It is, therefore, possible to prevent de novo methylation in response to exposure or learning and memory episodes by treating with site-specific dCas9-gRNA before the anticipated trigger. This treatment would sterically inhibit future de novo methylation. Removal of dCas9 or its turnover would then enable interaction between the unmethylated position and the transcription machinery and other factors in the future. Such an approach could help understand the role of site-specific de novo methylation in neuronal activation, stress responses, and other context-dependent processes.

Dynamic DNA methylation responses, such as those observed during neuronal activation, involve not only de novo methylation but also site-specific demethylation of specific CG positions in key genes [80,81]. Replication-independent demethylation is believed to occur through a sequence of oxidations of the methyl group on the cytosine base by TET enzymes followed by the removal of the oxidized methyl-cytosine through base excision repair [82,83]. Since site-specific demethylation relies on interaction with TET enzymes, it is also potentially amenable to inhibition via steric hindrance using gRNA-targeted dCas9. By applying steric hindrance prior to neuronal activation or other triggers, demethylation at the targeted CG site could similarly be prevented. Upon removal of dCas9 or its turnover, the methylated site could then interact with other factors once the transient demethylation trigger subsides. This would allow for the assessment of the functional role of site-specific demethylation by comparing the physiological and phenotypic responses of animals that retain methylation at the position and those that were demethylated in response to treatment. Although the inhibition of the TET enzyme interaction by gRNA-targeted dCas9 has not yet been tested, it is a promising approach that fundamentally parallels DNMT inhibition and could be optimized for studying site-specific demethylation in dynamic systems.

## 7. Concluding Remarks

The steric hindrance method offers a feasible approach to study the role of DNA methylation in dividing cells, including pluripotent and progenitor cells. By using steric hindrance, researchers can focus on the impact of specific sites and avoid the pitfalls of broad, off-target effects of dCas9-tethered epigenetic enzymes. The transitory nature of steric inhibition also allows researchers to study the effects of the loss of DNA methylation at defined time points during differentiation, aging, and other physiological processes on downstream events and methylation trajectories.

One interesting question that researchers can address using this system is the stability of the dCas9-induced loss of DNA methylation through subsequent stages of the life cycle. It is anticipated that signals existing in the cell or triggered during subsequent differentiation stages could restore the original methylation state. However, an alternative possibility is that transient demethylation by dCas9 could elicit downstream epigenetic modifications that stabilize the demethylated state. In our study [35], dCas9-induced demethylation was stable; however, a transitory DNA methylation loss that has been mostly restored by DNMTs has been seen in studies with DNA demethylation agent 5-aza-2′-deoxycytidine [83]. The long-term outcome of the methylation change is, thus, likely to be dependent on numerous factors, such as de novo methyltransferase activity, intrinsic, and extrinsic signals, and the context of other epigenetic marks in the region, all of which also vary with cell type. Although the loss of DNA methylation may not persist through subsequent stages of differentiation or the life cycle of the cell or animal, it might trigger downstream events which might have long-term impacts through intermediary steps. Therefore, studying the potential long-term downstream impact of transitory demethylation can provide valuable insights into understanding how epigenetic marks can interact across time and trigger downstream cascades of epigenetic changes.

Barring the development of new strategies to restrict the activity of dCas9-TET-based epigenetic editors to their intended targets, the use of dCas9-TET in causality research remains unreasonable and is obscured further by the methylation-independent activities of TET and by the fact that TET enzymes are dioxygenases that deposit unique epigenetic modifications. Nevertheless, if the goal of a study or a clinical intervention is to activate a gene stably and avoid rebound silencing, the use of TET-tethered dCas9 is beneficial, regardless of the epigenetic modification involved. Since steric hindrance might not provide the stability of a demethylated state achieved by (constitutive) expression of dCas9-TET, there may continue to be a need for dCas9-TET epigenetic editing as well as dCas9-based recruitment of other transcriptional activators, especially in a clinical setting.

The steric hindrance method cannot achieve silencing by methylation of an unmethylated site, thus necessitating the use of a dCas9-based DNMT editing tool [40]. However, as discussed, this inevitably triggers genome-wide and off-target changes in DNA methylation [46]. An alternative method that does not involve expressing an ectopic DNMT involves integrating CG-less floxed DNA into a CG island to trigger stable de novo methylation of the CG island [54]. However, this method has its limitations and potential confounders as discussed above, including the fact that it can only work in cells with high de novo methylation activity, it requires double-strand breaks, integration, and excision by recombination which might compromise genomic sequence integrity, it cannot be targeted to specific CGs (only large CG islands), and risks CRISPR/Cas9-based off-target mutation of the genome. Nevertheless, this method appears to induce DNA methylation that is stable through development and intergenerationally and has important research applications. Although the clinical utility of such an approach seems limited, it might have a role in cellular therapy where induced pluripotent stem cells from a patient are epi-edited in vitro and then transplanted back into the patient following differentiation into specific progenitors or terminally differentiated cell types.

Steric hindrance cannot induce loss of nondynamic DNA methylation in non-dividing, terminally differentiated cells, such as neurons or muscle cells. This limitation restricts its utility in addressing the role of site-specific methylation in fully differentiated systems and limits its clinical use. However, an agent that can cause site-specific demethylation in dividing cells still has a wide range of utility. One approach is to use ex vivo methods with either induced pluripotent cells or progenitor cells that can be epigenetically modified and reintroduced to the patient, such as the activation of insulin through trans-differentiation and epigenetic editing. Another approach is to use site-specific demethylating agents for cancer therapy which can demethylate and activate tumor suppressor genes while avoiding the demethylation of tumor-promoting and metastatic genes. Site-specific demethylation also has potential use in immunotherapy as differentiation and activation involve cell division.

Although interference with dynamic methylation–demethylation is of great value for research purposes and for understanding the role of site-specific dynamic methylation in development, differentiation, and physiological responses in the brain and other tissues, it is currently difficult to see how this could be utilized in the clinic, except for cellular therapy. However, once our understanding of the role of dynamic DNA methylation in different pathologies is further developed, possibly directly as a result of the utilization of the steric hindrance approach, such tools might have important clinical applications.

As the use of DNA methylation inhibitors in the treatment of various clinical conditions is becoming increasingly supported, the need for site-specific demethylation agents that can be administered in a clinical setting is growing. However, the limitation of current drugs is their non-specificity as they may target numerous methylation positions with the potential for consequential side effects. Targeted steric hindrance agents may address this need, but their advancement as clinical tools would require the use of proteins and RNA, rather than viral delivery and effective packaging to increase bioavailability and nuclear entry. This is currently a general challenge in the entire CRISPR field, and its advancement could turn steric hindrance into a potent pharmacological, site-specific demethylation agent.

## Data Availability

Not applicable.
